# Supplementary material for: Burden, predictors, and outcome of unconsciousness among under-five children hospitalized for community-acquired pneumonia: A retrospective study from a developing country
Source: PLoS One. 2023 Jun 21;18(6):e0287054. doi: 10.1371/journal.pone.0287054 (PMC10284379; doi:10.1371/journal.pone.0287054)
Supplement: Attachment — Submitted filename: Comments.docx [file pone.0287054.s002.docx]

**Major comments**

1. The authors mentioned unconsciousness among under-five children hospitalized for community-acquired pneumonia. Regarding unconsciousness, though the authors mentioned the percentage of pneumonia and severe pneumonia, however, the authors suggested stating how many of them developed severe pneumonia from pneumonia after admission (as it is a danger sign). It will provide a clear idea.
2. The inclusion and exclusion criteria for the study have to be clearly stated. For example, age 0-59 months or 2-59 months.
3. In L-166, the authors mentioned, data cleaning and corrections were done in cases of inconsistencies or errors in the entered data. Due to the retrospective nature of the data, how did they clean it up and fix the inconsistencies? It needs to explain clearly.
4. It's unclear why the authors included the ‘Hospital stay in days’ variable in Table 2. Better to provide an explanation.
5. In L-224-225, and L-284 the authors mentioned older children, but they did not mention the age group of the children before. Need to correct/revise it.
6. In Figure 2, the authors have shown only the months over the years. It is recommended to mention seasonality (monsoon, pre-winter, winter season) along with the month.
7. In L-290-292, the authors mentioned ‘Earlier motivation and health awareness of parents during the diagnosis of the congenital anomaly might compel them to seek health care early for their vulnerable children’. It would be better if they could give literature support/reference regarding this statement.
8. In L-298-300, the author could revise the sentences like this: The authors could write that the seasonality of pneumonia is known, but further study may be needed to determine the causes of the seasonality of unconsciousness in children with pneumonia.
9. In L-284-289, these sentences seem inconsistent with the study’s result. Please revise it.
10. In L-298, it is not about unconsciousness but also about pneumonia. Please rewrite the sentence.
11. Please add recent references (preferably the last 5-7 years) and follow the journal guidelines. The text citation would be [ ], not ( ). Please check the journal guidelines thoroughly.
12. The future recommendation is not strong. Please add a sentence about how these observations can be sustainable.

**Minor comments**

1. In L-41, in the first sentences, please add a citation.
2. In L-48, WHO needs to be abbreviated as it is used for the first time.
3. In L-84, please omit the word operated and replace it with conducted.
4. In L-86-88, the authors are suggested to omit this sentence.
5. In L-146, in oxygen words ‘O’ will be capitalized as the next word followed so. Please make it consistent.
6. In L-192-195, the authors are suggested to omit these sentences as this information was already mentioned in the Method section.
7. In Table 2, the authors need to mention whether the OR is unadjusted, as they mentioned in the rest of their tables. Please make it consistent.
8. The authors could add more points regarding strengths.
9. Please check the English grammar all over the manuscript.
