# Supplementary material for: Burden, predictors, and outcome of unconsciousness among under-five children hospitalized for community-acquired pneumonia: A retrospective study from a developing country
Source: PLoS One. 2023 Jun 21;18(6):e0287054. doi: 10.1371/journal.pone.0287054 (PMC10284379; doi:10.1371/journal.pone.0287054)
Supplement: Attachment — Submitted filename: Response to reviewers.docx [file pone.0287054.s003.docx]

**Rebuttal letter**

Reviewer #1: The authors primarily focused on the burden, predicting factors and outcome of unconsciousness among under five children hospitalized for community-acquired pneumonia, which I believe to be an important topic. The paper is interesting and generally well-written. I read the manuscript thoroughly, and my feedback as followed.

This is a retrospective case-control study, it is unclear that authors mention predicting factors instead of risk factors in title.

In line 48, it is unclear WHO. In line 55 to 60, authors find out predicting factors instead of risk factors in the retrospective case-control study. Author’s aim should identify the risk factors.

Authors miss the study site or country name in title and do not convey any situation of unconsciousness in their study areas in the introduction section. I found a typo mistake “ration” in line 77. This is a retrospective chart review from a historical record, it is not a match case control study. it is unclear the statement in line 77-78 “Though the case control ratio 1:10, …………………………………..not excluded the participants beyond 1:4 ratio.” I think that all eligible participants must be included in the study. Authors used chi-square test for examining the association between unconsciousness and pneumonia, but this test is not used to compare between two categorical variables (line # 171). In Data analysis section, authors unnecessarily mention chi-square test, Student’s t-test, and Mann-Whitney Test. Authors sometimes mention associated factors (line# 175) in the data analysis. Authors must select factors those were significant at level 10% for multivariate analysis. authors missed this point. In table 1, authors missed the odds ratio (OR) for age, Hemoglobin, and WBC count. Age, Hemoglobin, and WBC count are continuous but it is possible to calculate OR by using logistic regression. In line # 224, authors need to mention confounding variables. In Table 3, Multivariate regression analysis is used to adjust confounding variables in the relation between outcome and exposure. Authors used multiple logistic regression analysis to identify the risk factors of unconsciousness. All variables were adjusted for each other. It is unexpected to adjust hypoxemia, severe sepsis and so on for age and sex to estimate OR. It is good to adjust age and sex as confounding variables to estimate OR for hypoxemia or severe sepsis by using multivariate regression analysis. All results will be contradictory before fit best regression model. In conclusion, authors do not mention the preventable factors of unconsciousness.

**Response:**

Thank you for your kind reviews and valuable suggestions that helped us to improve the quality of this manuscript. We’ve modified the manuscript accordingly and addressed your important revisions as follows:

As in this retrospective case-control study, we tried to identify single or composite clinical features that can predict potential unconsciousness in a child with pneumonia/severe pneumonia, we used the term predicting factors instead of risk factors. We also admit that ‘predicting factors’ might not be a good term and thus we have replaced this with ‘predictors’ as the better term for this title and also in the manuscript (line # 55, 62, 65, 189, 251, 255 of the track change version).

Study site criteria is added in the title and the nearly related situation in the study country and also some related countries are added in the introduction (line # 56 to 61 of the track change version).

We have deducted the sentence related to case-control ratio to avoid any confusion among the readers. According to kind recommendation of the respected reviewer we have included all the eligible children in the study. So, the line with typo mistake is deleted (line # 85 and 86 of the track change version)

Reanalysis is done by logistic regression in case of chi-square test, t-test and Mann-Whitney test according to the reviewer’s suggestion (line # 182 to 188 of the track change version). It is important to mention that there are several recommended options for putting the variables in the regression model: popularly with those variables having 5% level of significance using chi-square test, t test and Mann-Whitney test and this can be extended up to 10% (O’Brien and Fleming). However, according to kind recommendation of the respected reviewer, data analysis plan is rewritten mentioning the inclusion of variables at 10% significant level in multivariate regression analysis (line # 192 and 193 of the track change version). Odds ratio (OR) and 95% CI level are also included in case of numerical variables (Table 1 of the track change version).

Confounding variables are mentioned under the statistical analysis section with justification for adding age and sex (line # 193 to 195 and 254 to 256 of the track change version).

Thanks, the respected reviewer to kindly suggest to include the preventable factors in the conclusion. We have incorporated this in our conclusion according to the recommendation of our respected reviewer (line # 347 to 349 of the track change version), although, conclusion stands alone, as it only allows us to write the summary of the results.

**Reviewer 2:**

**Thank you for your kind reviews and valuable suggestions that help us to improve the quality of this manuscript. We’ve modified the manuscript accordingly and addressed your important revisions as follows:**

**Major comments**

1. The authors mentioned unconsciousness among under-five children hospitalized for community-acquired pneumonia. Regarding unconsciousness, though the authors mentioned the percentage of pneumonia and severe pneumonia, however, the authors suggested stating how many of them developed severe pneumonia from pneumonia after admission (as it is a danger sign). It will provide a clear idea.

Response: As this is a retrospective analysis, we couldn’t collect the data of pneumonia participants who deteriorated to severe pneumonia. It has been added under limitations (Line- 338-339 of the track change version). Hope that the reviewer will consider this.

2. The inclusion and exclusion criteria for the study have to be clearly stated. For example, age 0-59 months or 2-59 months.

Response: Inclusion and exclusion criteria are stated more clearly under the study design (Line- 75 and 84 of the track change version).

3. In L-166, the authors mentioned, data cleaning and corrections were done in cases of inconsistencies or errors in the entered data. Due to the retrospective nature of the data, how did they clean it up and fix the inconsistencies? It needs to explain clearly.

Response: Explained in Line 178-180 (of the track change version).

4. It's unclear why the authors included the ‘Hospital stay in days’ variable in Table 2. Better to provide an explanation.

Response: Explained in Line 239-241 (of the track change version).

5. In L-224-225, and L-284 the authors mentioned older children, but they did not mention the age group of the children before. Need to correct/revise it.

Response: the line has been revised (Line- 30 and 247 of the track change version).

6. In Figure 2, the authors have shown only the months over the years. It is recommended to mention seasonality (monsoon, pre-winter, winter season) along with the month.

Response: seasonality has been mentioned in line 264-265 (of the track change version).

7. In L-290-292, the authors mentioned ‘Earlier motivation and health awareness of parents during the diagnosis of the congenital anomaly might compel them to seek health care early for their vulnerable children’. It would be better if they could give literature support/reference regarding this statement.

Response: some references of related studies regarding the parents’ concern about their child having congenital anomaly have been mentioned later in line 321-324 (of the track change version).

8. In L-298-300, the author could revise the sentences like this: The authors could write that the seasonality of pneumonia is known, but further study may be needed to determine the causes of the seasonality of unconsciousness in children with pneumonia.

Response: Revised accordingly (Line- 331-332 of the track change version).

9. In L-284-289, these sentences seem inconsistent with the study’s result. Please revise it.

Response: This finding is not the same as the mentioned studies as we’ve not found any study among unconsciousness in pneumonia patients. The lines are revised accordingly for better clarification (Line- 313-318 of the track change version).

10. In L-298, it is not about unconsciousness but also about pneumonia. Please rewrite the sentence.

Response: Rewritten accordingly (Line- 327 of the track change version).

11. Please add recent references (preferably the last 5-7 years) and follow the journal guidelines. The text citation would be [ ], not ( ). Please check the journal guidelines thoroughly.

Response: New recent reference is added (reference- 9 of the track change version) and referencing format is changed according to the reviewer’s suggestions throughout the manuscript.

12. The future recommendation is not strong. Please add a sentence about how these observations can be sustainable.

Response: Added under conclusions (Line 352-355 of the track change version).

**Minor comments**

1. In L-41, in the first sentences, please add a citation.

Response: citation added (L-42).

2. In L-48, WHO needs to be abbreviated as it is used for the first time.

Response: Elaborated accordingly (L- 49-50).

3. In L-84, please omit the word operated and replace it with conducted.

Response: Omitted (L-93).

4. In L-86-88, the authors are suggested to omit this sentence.

Response: Omitted (L- 95-97).

5. In L-146, in oxygen words ‘O’ will be capitalized as the next word followed so. Please make it consistent.

Response: Capitalized and made it consistent (L-156).

6. In L-192-195, the authors are suggested to omit these sentences as this information was already mentioned in the Method section.

Response: Thanks for your logical suggestions. We have omitted the results part from the methods and revised the methods section accordingly (L- 77-81). As this should better fit in the results section, we have retained this part in the results section.

7. In Table 2, the authors need to mention whether the OR is unadjusted, as they mentioned in the rest of their tables. Please make it consistent.

Response: Mentioned accordingly and made it consistent (Table 2).

8. The authors could add more points regarding strengths.

Response: More points are added (L- 340-344).

9. Please check the English grammar all over the manuscript.

Response: Thank you for your suggestion. We’ve checked throughout the manuscript and corrected accordingly.
